# Retraction Note: Genomic amplification and high expression of EGFR are key targetable oncogenic events in malignant peripheral nerve sheath tumor

**DOI:** 10.1186/s13045-022-01380-7

**Published:** 2022-10-27

**Authors:** Xiaoling Du, Jilong Yang, Antti Ylipää, Ze Zhu

**Affiliations:** 1 Department of Diagnostics, Tianjin Medical University, Tianjin, 300060 China; 2 Department of Bone and Soft Tissue Tumor, National Clinical Cancer Research Center, Tianjin Medical University Cancer Institute & Hospital, Tianjin, 300060 China; 3 Department of Pathology, The University of Texas MD Anderson Cancer Center, Houston, TX 77030 USA; 4 Department of Signal Processing, Tampere University of Technology, 33101 Tampere, Finland; 5 Department of Medical Microbiology, Tianjin Medical University, Tianjin, 300060 China

## Retraction to: Journal of Hematology & Oncology 2013, 6:93 10.1186/1756-8722-6-93

The Editor-in-Chief has retracted this article because of substantial text overlap with a previously published article by some of the same authors (1) and apparent re-use of some of the data shown in the figures with different labelling. Specifically:

- In Fig. 4a some Western blots appear to be very similar or to overlap with those presented in Fig. 3A in (1).
- Figure 4c appears to contain an image that overlaps with Fig. 3B in (1). Both images are described as “ST88-14 cell line, control siRNA”; however, the two figures purport to describe separate and different experiments.

Corresponding author Yang has confirmed that both articles are based on the same patient population and sample collection. Although the earlier study [1] is cited in the 2013 article, it was not made clear that this was a continuation of the same work by a subset of authors. Corresponding author Yang has stated that the original data are not available.

Dr. Jilong Yang does not agree to this retraction. The publisher was not able to obtain a current email address for Antti Ylipää. None of the other authors has responded to any correspondence from the Editor or publisher about this retraction.
